# ^15 ^N Reaction Monitoring at Low and Inhomogeneous Magnetic Fields Enabled by Hyperpolarization with Parahydrogen

**DOI:** 10.1002/chem.202503018

**Published:** 2025-11-04

**Authors:** Gonzalo Gabriel Rodriguez, Ruhuai Mei, Lisa Maria Fries, Stefan Glöggler

**Affiliations:** ^1^ NMR Signal Enhancement Group Max Planck Institute for Multidisciplinary Sciences Am Fassberg 11 37077 Göttingen Germany; ^2^ Center for Biostructural Imaging of Neurodegeneration University Medical Center Göttingen Von‐Siebold‐Str. 3A 37075 Göttigen Germany; ^3^ Advanced Imaging Research Center University of Texas Southwestern Medical Center 5723 Forest Park Rd TX75390 USA

**Keywords:** ^15 ^N chemical reaction monitoring, hyperpolarized ^15^N, inhomogeneous fields, low field, PHIP

## Abstract

The combination of hyperpolarization methods with low and inhomogeneous fields promises to expand the application of nuclear magnetic resonance (NMR) to fields that nowadays are not standard. Reducing the magnetic field strengths and homogeneity requirements opens the door to a wide variety of magnet designs that can allow for monitoring chemical reactions in the benchtop or even larger reactors. In this work, we propose a method to monitor ^15 ^N chemical reactions at low and inhomogeneous fields based on parahydrogen‐induced polarization. The concept was demonstrated at a magnetic field of 66 mT and 81 ppm inhomogeneity. Specifically, we measured the conversion of ^15^N‐boronobenzyl‐2‐styrylpyridinium (^15^N‐BBSP) to ^15^N‐2‐styrylpyridin (^15^N‐SP). This enables the detection of H_2_O_2_ with high sensitivity and selectivity, exhibiting a significant chemical shift difference of 88.4 ppm, when ^15^N‐SP is obtained after a base‐assisted 1,6 elimination/rearrangement. The long T_1_ of ^15^N‐BBSP at low magnetic fields (194 s at 100 mT) exemplifies the potential of hyperpolarized ^15 ^N for low field applications. We envision the implementation of our methods for studying chemical reactions in benchtop devices, drug screening, investigating biological systems in their native environments, preclinical research and contrast development for low‐field and portable magnetic resonance imaging (MRI).

## Graphical abstract

^15 ^N reaction monitoring at low and inhomogeneous fields enabled by PHIP. The concept was demonstrated at a magnetic field of 66 mT and 81 ppm inhomogeneity. Specifically, we measured the conversion of ^15^N‐boronobenzyl‐2‐styrylpyridinium to ^15^N‐2‐styrylpyridin. This enables the detection of H_2_O_2_ with high sensitivity and selectivity, exhibiting a significant chemical shift difference of 88.4 ppm.

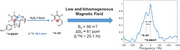

## 1 Introduction

Hyperpolarization is a technique that can provide enhancements of several orders of magnitude of nuclear magnetic resonance (NMR) signals.^[^ ^1^ ^]^ This dramatically boost of sensitivity has been widely implemented across multiple disciplines. Hyperpolarization plays a key role in chemical characterization, reaction monitoring, studying catalytic cycles,^[^ ^2^ ^]^ and biological in vitro/cell studies^[^ ^3^ ^]^ and preclinical research.^[^ ^4^ ^]^ Recently, hyperpolarization methods have generated excitement in the clinical community because it enables visualization of metabolic fluxes and disease‐encoding biomolecules in the shape of magnetic resonance imagining (MRI).^[^ ^5^ ^]^ However, despite the significant signal amplification provided by hyperpolarization, most reported studies still rely on high‐field and homogeneous NMR spectrometers and MRI scanners (B_0_  1 T & ΔB_0_ 10 ppm). This is largely because spectral dispersion, chemical shift referencing, and quantification are simpler and more robust at high field. Nevertheless, this reliance on high‐field and homogeneous devices imposes several limitations, including high cost; difficulty studying reactions within metallic chambers; challenges with spectroscopy in the presence of ferromagnetic and paramagnetic substances^[^ ^6^ ^]^; impediments to benchtop studies; restrictions on investigating the metabolism of biological systems in their native environments and in cell culture rooms, and, moreover, the translation and accessibility of metabolic imaging to the clinics. Therefore, reducing the requirements of the main magnetic field decreases the initial and maintenance cost of the devices, and more importantly, allows to explore a wide range of magnet designs that can derive on lightweight, portable, compact, and benchtop devices and tackle the challenges previously mentioned for high‐magnetic fields. Consequently, the combination of hyperpolarization with low‐field devices (B_0_  1 T) has the potential to extend NMR and MRI applications to fields that were never explored before. As a simple example, it would be possible to design scanners where it is not necessary to remove cell cultures from their original flask and to place such a scanner into a cell culture room with demanding biosafety requirements, allowing for metabolism investigation without altering their native environment and minimizing cells stress.

Among the hyperpolarization approaches, Parahydrogen‐induced polarization (PHIP)^[^ ^7^ ^]^ is a rapid approach of hyperpolarization and can be included in lab settings, larger reactors and imaging centers. PHIP is gaining international attention due to its simplicity and affordability as a hyperpolarization method. Significant recent advances include signal amplification by reversible exchange (SABRE),^[^ ^8^ ^]^ p‐H_2_‐induced polarization with side‐arm hydrogenation (PHIP‐SAH) to enhance the signals of metabolites,^[^ ^4^ ^]^ and rapid hyperpolarization and purification techniques.^[^ ^9^ ^]^ These advances demonstrated the feasibility of producing hyperpolarized agents through portable, cost‐effective approaches.^[^ ^10^ ^]^ Since p‐H_2_ can be produced using simple tools, such as cooling hydrogen gas with liquid nitrogen, these innovations could offer MR sensitization capabilities in settings where high costs or a lack of infrastructure have traditionally been limiting factors. Consequently, the emergence of low‐cost, portable hyperpolarization devices has the potential to make advanced NMR including analytics, drug screening and, metabolic imaging more accessible for a wide range of applications.

Nevertheless, despite the significant simplification offered by PHIP, as mentioned above, the initial installation and ongoing maintenance costs of the NMR spectrometers and MRI scanners remain a major obstacle to widespread accessibility and implementation of these techniques beyond their standard conditions. In this regard, the MRI and NMR communities had been exploring different approaches toward the mitigating these limitations. In the MRI community, it has been a considerable trend toward developing MRI scanners that operate at low or intermediate magnetic fields (1 mT < B_0_ < 1 T).^[^ ^11^ ^]^ These scanners can consist of either electromagnets^[^ ^12^ ^]^ or permanent magnets^[^ ^13^ ^]^ to reduce technological complexity and cost. However, most of these prototypes focus only on ^1^H MRI limiting the implementation of hyperpolarization experiments, with only one device having multinuclear capabilities for humans^[^ ^14^ ^]^ and a few for preclinical studies.^[^ ^15^ ^]^ On the other hand, the NMR community has focused on zero to ultra‐low‐field (ZULF) NMR (B_0_ < 1 mT).^[^ ^16^ ^]^ This approach requires compensation for external magnetic fields, such as the Earth's field and additional laboratory fields. In ZULF NMR, Larmor frequencies fall within the audio range. This allows nuclear spins to be manipulated using direct current (DC) magnetic field pulses. Highly sensitive magnetometers are employed for detection. At these low magnetic fields, chemical shift effects are not observable, and the spins are in the strong coupling regime. Therefore, ZULF NMR spectra primarily reflect pure J‐coupling information. ZULF NMR has successfully demonstrated its ability to monitor chemical and enzymatic reactions using hyperpolarized ^13^C,^[^ ^17^, ^18^ ^]^ as well as chemical exchange in hyperpolarized ^13^C and ^15 ^N compounds.^[^ ^18^, ^19^ ^]^ However, to the best of our knowledge, time resolved monitoring of ^15 ^N labelled reactants using PHIP at zero‐ to ultraslow‐field has not yet been demonstrated.

^1^⁵N has a highly informative chemical shift range of 1,350 ppm and a long T_1_ relaxation time.^[^ ^20^ ^]^ However, since ^15 ^N is only 0.36% naturally abundant and has a gyromagnetic ratio (γ) that is ten times smaller than ^1^H, it is approximately 260000 times more difficult to detect. Importantly, the ^15 ^N isotope can be sourced inexpensively from materials such as ^15^NH_4_Cl and Na^15^NO_3_, which provide accessible routes for synthesizing other isotopically labeled compounds, including pharmaceuticals. The sensitivity gains offered by PHIP have been widely exploited to facilitate the study of organic and inorganic compounds and enable the detection of previously unobservable intermediates,^[^ ^21^, ^22^, ^23^, ^24^, ^25^, ^26^, ^27^ ^]^ with studies even showing high quality spectral resolution at milli‐Tesla fields.^[^ ^28^ ^]^ In this work, we aim to demonstrate that chemical reactivity can be tracked at low magnetic fields, and that low field ^15 ^N hyperpolarization can be expanded to encompass greater diversity of applications.

Here, we present the first demonstration of chemical reaction monitoring using ^15 ^N hyperpolarization at low and inhomogeneous magnetic fields. NMR spectra were acquired at 66 mT, with an approximate B_0_ inhomogeneity (ΔB_0_) of 81 ppm. This field strength is sufficient to reveal observable chemical shift effects and does not require the compensation of the external magnetic fields. This field range, combined with the relaxed homogeneity requirements, allows us to envision the implementation of our method across a variety of magnet designs. As a result, a broad range of applications becomes feasible, including low‐field human MRI, benchtop devices for reaction monitoring for, for example, pharmaceutical production, and studies of cells within their native environments. We furthermore demonstrate the proof of concept for detecting reactions of ^15 ^N containing compounds at millitesla fields by monitoring a reactive oxygen sensitive probe, a chemical species relevant in cancer and its treatment.^[^ ^29^, ^30^ ^]^

## 2 Results and Discussion

### 2.1 Low‐Field System and ^15 ^N Detection

**Figure 1:**
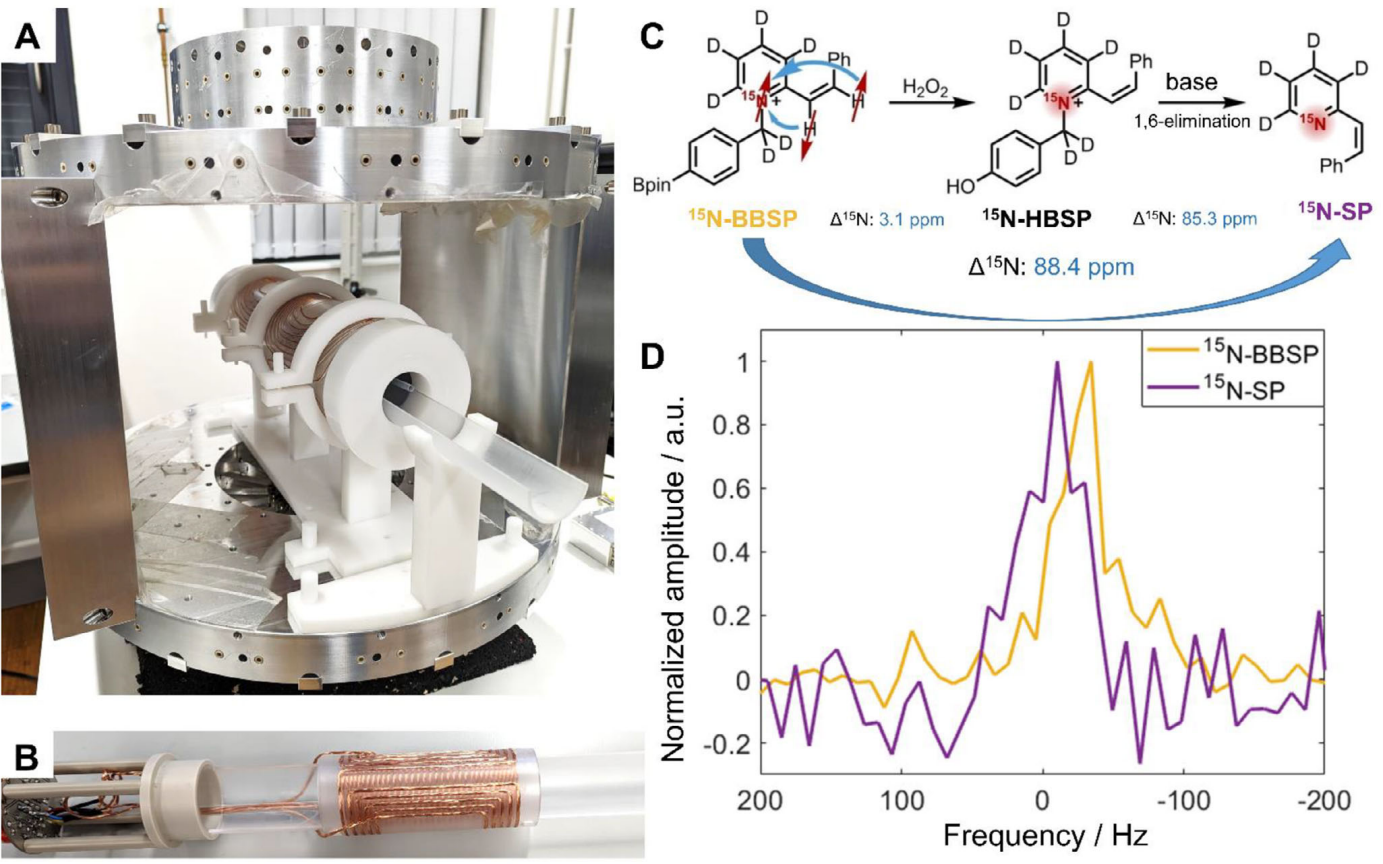
Low‐field system built in‐house with capabilities for ^15 ^N detection. A) A four rings array main magnet together with the assembly of all the coils (3 gradient, 2 shimming and RF multinuclear coil). B) RF multinuclear coil. The coil is composed by the five‐turn saddle coil that operates at ^1^H frequency and the variable gap solenoid that is used for detection of ^15 ^N, ^23^Na, and ^13^C. C) Schematic of the reaction chain, a chemical shift of 88.4 ppm is expected between the ^15^N‐BBSP and ^15^N‐SP (adapted from reference [31] copyright license ID 1 660 582–1). D) NMR spectra of hyperpolarized ^15^N‐BBSP and ^15^N‐SP acquired at 66 mT. The precursor for both samples was ^15^N‐BBPEP (200 µL, 50 mM) with 10 mM catalyst.

The low‐field experiments were performed in our 66 mT multinuclear scanner. This device was built in‐house and introduced by Rodriguez et al.^[^ ^13^ ^]^ Here, we designed the radiofrequency (RF) coil and sequence parameters to be able to detect ^15 ^N spins in addition to ^23^Na and ^13^C. Specifically, we introduced a matching and tuning circuit that allows to select the resonance frequencies for each X‐nuclei by changing the position of an analogic switch, and, calibrated the pulse length and amplitude. The scanner consists of a main magnet design based on a 4‐rings array with axially directed magnetization and mirror symmetry with a 28 cm gap composed by 64 neodymium magnets, contained in an aluminum frame. As a result, the main magnet only weights 83 kg, enabling portability. The simulated peak‐to‐peak inhomogeneity in a full cylinder of 100 mm length and 40 mm diameter placed in the geometrical center of the magnet is 2796 ppm. Remarkably, with the incorporation of only three linear gradients and two shimming coils, it was possible to obtain sufficient field homogeneity and hence enough spectral resolution to resolve the chemical shift of 81 ppm. Figure 1A shows the main magnet together with the assembly of all the coils. Figure 1B shows the multinuclear RF coil for the detection of ^15 ^N at 66 mT. Details on the magnet, calibration and shimming can be found in references.^[^ ^15^ ^]^

Given the low gyromagnetic ratio of ^15 ^N and the low acquisition field, the use of hyperpolarization approaches to enhance the NMR signals is required to detect ^15 ^N signals. To demonstrate the feasibility of reaction monitoring, we used our recently introduced ^15^N‐heterocycle ^15^N‐boronobenzyl‐2‐styrylpyridinium (^15^N‐BBSP).^[^ ^28^ ^]^ It is a sensitive probe for reactive oxygen species (ROS) such as hydrogen peroxide and such species are, for example, relevant for the detection of treatment response of cancer. The ^15 ^N spins show a significant chemical shift difference of up to 88.4 ppm, when ^15^N‐2‐styrylpyridin (^15^N‐SP) is obtained in the presence of reactive oxygen and after a base‐assisted 1,6 elimination/rearrangement.^[^ ^31^ ^]^ Remarkably, the side‐arm cleavage of ^15^N‐BBSP is not possible without the incorporation of H_2_O_2_ and the intermediate formation of ^15^N‐hydroxybenzyl‐2‐styrylpyridinium (^15^N‐HBSP). Figure 1C shows the chemical reaction scheme. Hyperpolarized ^15^N‐BBSP has already been detected in vivo, and its capability to sense H_2_O_2_ opens the door to a diverse range of biomedical applications.

At first, we investigated the performance of the ^15 ^N coil and detected the hyperpolarized signal of ^15^N‐BBSP. The hyperpolarization protocol for ^15^N‐BBSP is as follows^[^ ^31^ ^]^: ^15^N‐boronobenzyl‐2‐phenylethynylpyridinium (^15^N‐BBPEP) was hydrogenated with pH_2_ at 310 K in MeOD‐d_4_ using the homogeneous rhodium catalyst [Rh(dppb)(COD)][BF_4_] (dppb: diphenylphosphino butane, COD: cyclooctadiene). In the ^1^H polarization step, 16.9 ± 0.9% polarization was achieved with three parallel experiments by comparison with the thermal spectrum. Subsequently, ^15 ^N polarization was realized by utilizing the MINERVA (Maximizing Insensitive Nuclei Enhancement Reached Via parahydrogen Amplification) sequence^[^ ^6^ ^]^ with a final polarization of 5.1 ± 0.4%. The yellow spectrum of Figure 1D shows the ^15 ^N signal from hyperpolarized ^15^N‐BBSP for a 50 mM precursor and 10 mM catalyst concentration in a 200 µL solution of methanol at 66 mT. Once ^15^N‐BBSP was detected, we proceeded to the detection of 15N‐SP. Thus, we added Na_2_CO_3_ and H_2_O_2_ to cleave all the ^15^N‐BBSP. The purple spectrum of Figure 1D shows the ^15 ^N signal of hyperpolarized ^15^N‐SP after correcting for thermal drifts. The measured peak difference between the ^15^N‐BBSP and ^15^N‐SP spectra is 24 Hz, which is slightly deviated from the 25.1 Hz expected from the 88.4 ppm chemical shift. This small discrepancy can be due to the changes in the solvent after the addition of base and H_2_0_2_, which modifies the pH. Therefore, to avoid this point and prove that we can monitor the reaction, we continued to detect a mixed state where both molecules coexist in the same solvent as described in the following section.

### 2.2 Reaction Monitoring at Low and Inhomogeneous Fields

**Figure 2:**
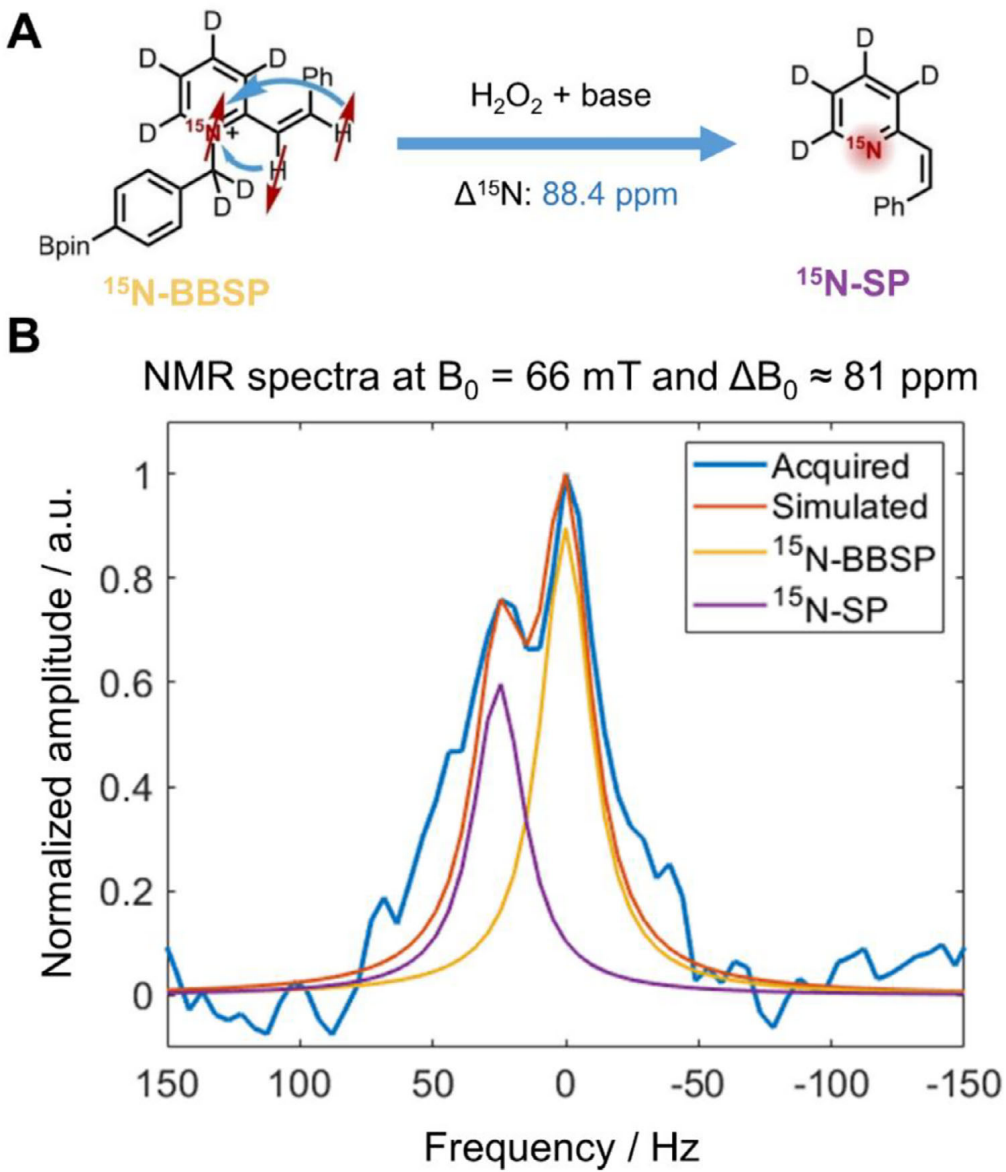
Reaction monitoring of hyperpolarized ^15^N‐BBSP at low and inhomogeneous magnetic fields. A) Schematic of the reaction chain, a chemical shift of 88.4 ppm is expected between the ^15^N‐BBSP and ^15^N‐SP after the addition of H_2_O_2_ and base (adapted from reference [31] copyright license ID 1 660 582–1). B) Acquired and simulated ^15 ^N NMR spectra for a 1 equivalent H_2_O_2_ and 50 mM base injection at 66 mT and 81 ppm field inhomogeneity.

With the established achievability to detect the ^15 ^N signal of the shown chemical probe, we investigated the feasibility of reaction monitoring (Figure 2A). The reaction conditions were set to be sufficiently slow in order to monitor the reactant and product peak at the same time in order to prove the feasibility to identify two different chemical species. This means that we adjusted the H_2_O_2_ and base concentration in such a way that both ^15^N‐BBSP and ^15^N‐SP compounds coexist. Specifically, we hyperpolarized a 50 mM precursor concentration in a 200 µL solution of methanol, followed by a 50 µL injection of D_2_O solution with Na_2_CO_3_ (50 mM) and H_2_O_2_ (200 mM, 1.0 equiv) concentrations. Figure 2B shows the ^15 ^N NMR spectrum for H_2_O_2_.

Then, the concentration of ^15^N‐BBSP and ^15^N‐SP together with the estimated ΔB_0_ were obtained from a fingerprint matching. The dictionary was generated by the addition of two individual Lorentz functions separated by 25.1 Hz (equivalent to 88.4 ppm at 66 mT). The inhomogeneity was simulated as the full width half maximum of each Lorentzian with ΔB_0_ values between 10 and 30 Hz in steps of 1 Hz. Finally, the two functions were added for concentration values ranging from 0 to 100% in steps of 2.5%. This resulted in a dictionary with a total of 861 entries. The matching process consists of two steps. First, the 40 spectra with the highest correlation with the acquired data were selected, which corresponds to the highest 5% spectra from the dictionary. Subsequently, the best spectrum was obtained by minimizing the peak intensity difference between the acquired and simulated data. The two‐step fingerprint matching strategy showed higher robustness under noisy data, given that the spectrum with the highest correlation does not necessarily yield the best matching.^[^ ^32^ ^]^

Figure S1, in the supplementary information, shows another two spectra acquired with 1 equivalent H_2_O_2_ and one spectrum with 2.7 equivalents (540 mM). In all the experiments, it was possible to observe two peaks, allowing to differentiate between ^15^N‐BBSP and ^15^N‐SP. The results from the fingerprint matching were: 60% ^15^N‐BBSP concentration and 23 Hz inhomogeneity with a correlation value of 0.9615 for the spectra shown in Figure 2B. 52.5% ^15^N‐BBSP concentration and 22 Hz inhomogeneity with a correlation value of 0.9376 for the spectra shown in Figure S1A. 70% ^15^N‐BBSP concentration and 14 Hz inhomogeneity with a correlation value of 0.8940 for the spectra shown in Figure S1B. 40% ^15^N‐BBSP concentration and 25 Hz inhomogeneity (81 ppm) with a correlation value of 0.8938 for the spectra shown in Figure 1C, which corresponds to 2.7 H_2_O_2_ equivalents.

The correlation values were higher than 0.89 for the four datasets, which demonstrates the robustness of the method. The inhomogeneity values were between 14 and 25 Hz. This variability is mainly associated with the placement of the NMR tube in different positions with respect to the magnet and it can be addressed with the construction of a more precise sample holder. The 2.7 equivalent experiment (Figure S1C) showed lower ^15^N‐BBSP concentration, which is consistent with the expected result. The 1 equivalent H_2_O_2_ experiments showed a variability between the calculated ^15^N‐BBSP concentrations in the range of 52.5% and 70%. The differences between the calculated concentrations are attributed to time variations between the H_2_O_2_ injection and the beginning of the acquisition of the NMR spectra, which were approximately 3 ± 2 s. The conversion from ^15^N‐BBSP and ^15^N‐SP involves dynamic rates that are in the range of this time scale and can affect the calculated value, as demonstrated at high magnetic fields in the following section.

### 2.3 High Field Dynamic Monitoring and Relaxation

**Figure 3:**
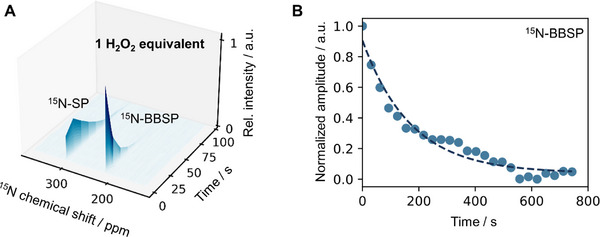
Dynamic reaction monitoring of hyperpolarized ^15^N‐BBSP at high field and T_1_ relaxation measurement. A) ^15^N‐SP and ^15^N‐BBSP dynamic acquisition after addition of H_2_O_2_ (2.7 equiv.) and Na_2_CO_3_ (25 mM). The initial solution was a 200 µL deuterated methanol (d_4_) solution with 2.5 mM precursor molecule and the spectra were measured at 7 T. A chemical shift of 88.4 ppm is measured between the ^15^N‐BBSP and ^15^N‐SP. B) T_1_ measurement of ^15^N‐BBSP in a deuterated methanol (d_4_) solution at 100 mT. The relaxation time was measure with field‐cycling and the signals acquired at 7 T.

Dynamic monitoring of the ^15^N‐BBSP to ^15^N‐SP reaction was performed at 7 T to provide a deeper understanding of the dynamics involved. Specifically, a ^15 ^N spectrum was acquired every 20 s after the application of an 18° flip angle. Figure 3A shows the dynamic spectra acquired for the ^15^N‐BBSP reaction of 200 µL methanol solution with 2.5 mM precursor concentration followed by a 50 µL injection of a D_2_O solution with Na_2_CO_3_ 25 mM and 1 equivalent of H_2_O_2_ (10 mM. 1.0 equiv). Additionally, Figure S2A, in the supplementary information, shows the results from the dynamic acquisition for H_2_O_2_ (15 mM. 1.5 equiv).

As it is possible to observe in Figure 3A, the initial signal from ^15^N‐BBSP is higher than the ^15^N‐SP and rapidly decreases. Specifically, for t = 0 s (first acquisition), the relationships between the two peaks gives a concentration of 80.1% for the ^15^N‐BBSP, while for t = 20 s (second acquisition) it is 45.3%. This is consistent with the results obtained from the low field experiments that showed variabilities between 52.5% and 70%, and are contained within the 80.1% to 45.3% range. Similar behavior is observed for 2.7 equivalents H_2_O_2_, with fast changes in ^15^N‐BBSP signal in the first 20 s (see Figure S2A). Additionally, the longitudinal relaxation time T_1_ of the compounds were measured. The measurements involved field cycling experiments for a magnetic field of 100 mT and the signals were acquired at 7 T as recently described.^[^ ^31^ ^]^ Figure 3B shows the measurements for ^15^N‐BBSP in methanol, resulting in a T_1_ = 194 ± 1 s. Figure S2B, in the supplementary material, shows the measurement for ^15^N‐SP in methanol, resulting in a T_1_ = 45 ± 6 s. It is worth highlighting that to obtain 100% ^15^N‐SP it was necessary to add high concentrations of H_2_O_2_ (62 equiv) which can affect the measured T_1_. Moreover, the T_1_ of ^15^N‐BBSP was also measured for 100 mT for H_2_O and D_2_O solvents with T_1_ = 133 ± 13 s for D_2_O and T_1_ = 77 ± 6 s for H_2_O (see Figure S3 in the supplementary information). The T_1_ value measured at 100 mT in methanol is 4.8 times longer than the reported at 7 T for the same solvent,^[^ ^25^ ^]^ demonstrating the big potential of hyperpolarized ^15 ^N at low magnetic fields. We decided to measure T_1_ at 100 mT instead of exactly 66 mT due to two main reasons. First, it allows us to compare the relaxation time to other probes previously developed in our lab.^[^ ^33^ ^]^ Second, we are measuring the relaxation through field cycling at our high field scanner and the field decays with the square of the distance, leading to a volume region for 66 mT that is smaller than for 100 mT and therefore the uncertainty of the measurement is higher.

## 3 Conclusion

We delivered the proof of concept that chemical reactions of ^15 ^N compounds can be monitored at low and inhomogeneous fields by employing PHIP hyperpolarization methods a. The minimum requirements for the magnetic field intensity and homogeneity open the doors for multiple applications for hyperpolarized ^15 ^N. The use of low or intermediate magnetic fields (1 mT < B_0_ < 1 T) represents a key advantage with respect to ZULF (B_0_ < 1 mT) because it allows to exploit the big chemical shift range of ^15 ^N for monitoring chemical reactions while keeping the hardware requirements low. We envision the development of dedicated scanners tailored for specific applications. Examples include compact designs for the implementation of our methods for monitoring chemical reactions in benchtop devices, drug screening, in vitro diagnosis, as well as, designs with big magnet bores for investigating biological systems in their native environments, preclinical research and contrast development for low‐field and portable MRI. Furthermore, the possibility to operate with inhomogeneous fields opens the doors for the implementation of this methodology to investigate conversions that take place in larger reactors.

## Supporting Information

Figures S1, S2, and S3 can be found within the Supporting Information.

## Conflict of Interest

The authors declare no conflict of interest.

## Supporting information



Supporting Information

## Data Availability

The data that support the findings of this study are available from the corresponding author upon reasonable request.
