# Supplementary material for: 15 N Reaction Monitoring at Low and Inhomogeneous Magnetic Fields Enabled by Hyperpolarization with Parahydrogen
Source: Chemistry. 2025 Nov 4;31(69):e03018. doi: 10.1002/chem.202503018 (PMC12699185; doi:10.1002/chem.202503018)

## Supplementary information

## 1. $^{15}N$ Reaction Monitoring at Low Magnetic Fields.

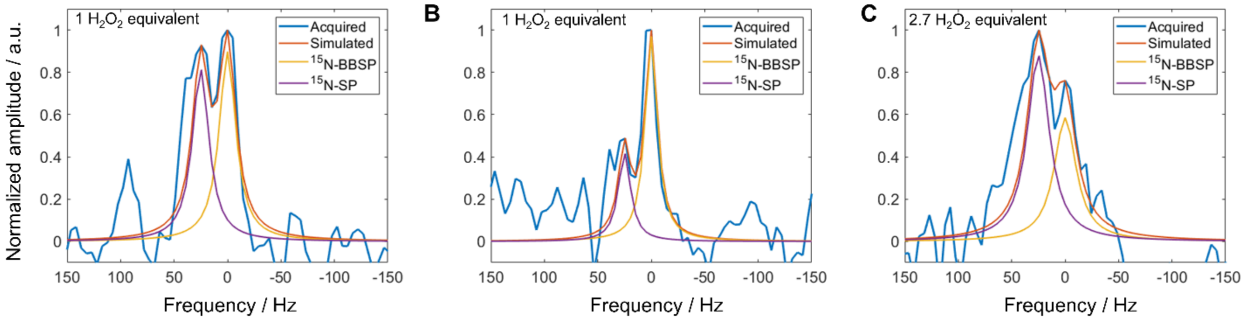


**Figure S1.** Reaction monitoring of hyperpolarized $^{15}N$-BBSP at low and inhomogeneous magnetic fields. A & B) Acquired and simulated $^{15}N$ NMR spectra for a 1 equivalent $H_2O_2$ injection. C) Acquired and simulated $^{15}N$ NMR spectra for a 2.7 equivalent $H_2O_2$ injection.

## 2. Dynamic Reaction Monitoring at High Field and Relaxation Measurements

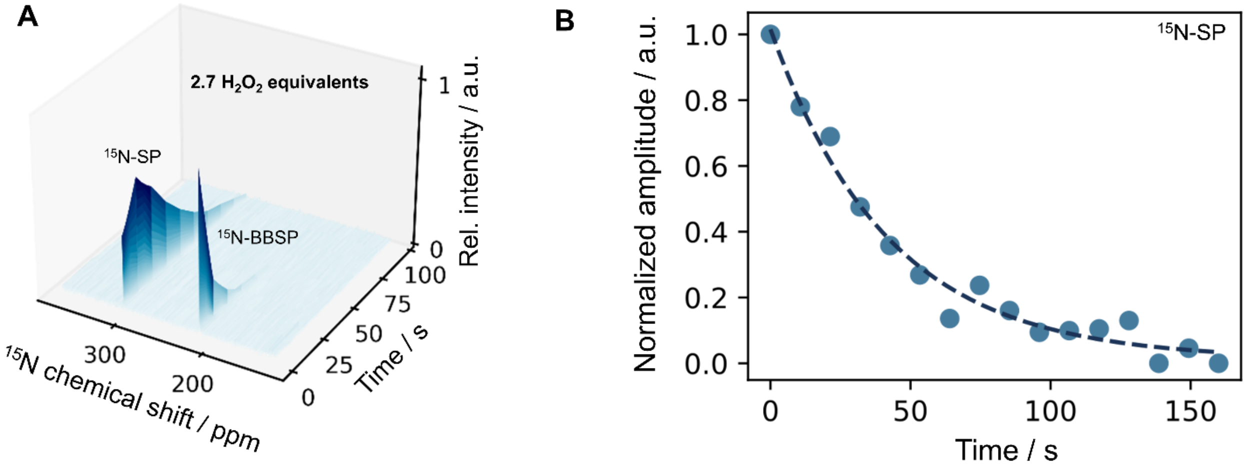


**Figure S2.** Dynamic reaction monitoring of hyperpolarized $^{15}N$-BBSP at high field and $T_1$ relaxation measurement. A) $^{15}N$-SP and $^{15}N$-BBSP dynamic acquisition for 2.7 $H_2O_2$ equivalents. A chemical shift of 88.4 ppm is measured between the $^{15}N$-BBSP and $^{15}N$-SP. B) $T_1$ measurement of $^{15}N$-SP in a methanol solution.

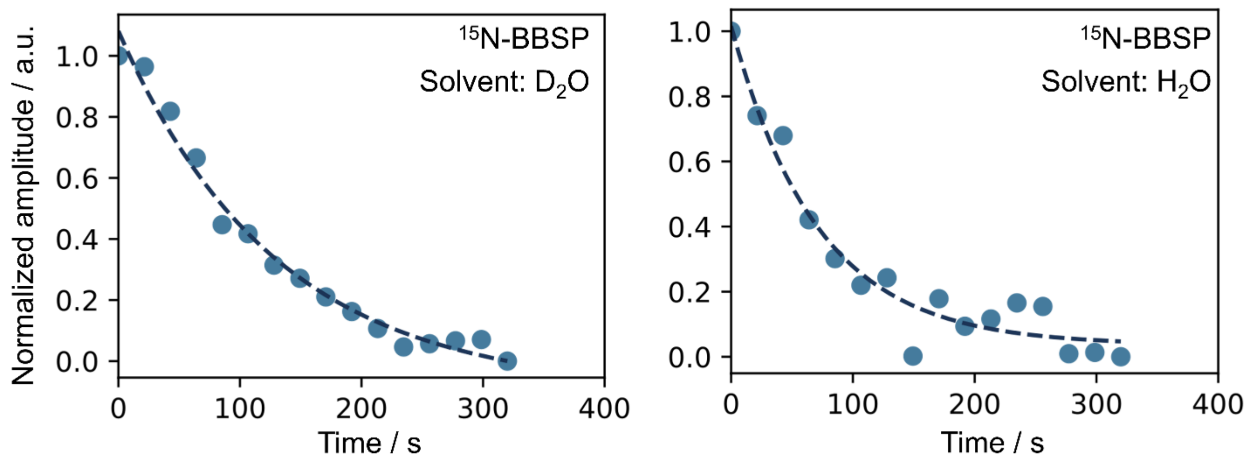


**Figure S3.** Longitudinal relaxation time measurements of. $^{15}$N-BBSP in different solvents. A) $^{15}$N-BBSP in $D_2O$. B) $^{15}$N-BBSP in $H_2O$.

## 3. Synthetic procedures for $^{14}$N-BBSP and its precursor $^{14}$N-BBPEP

### 3.1 General remarks

Chemicals were purchased from commercial suppliers and were used without further purification. 2-(Phenylethynyl)pyridine,[1] 2-(4-(bromomethyl)phenyl)-4,4,5,5-tetramethyl-1,3,2-dioxaborolane, [2] and (Z)-2-styrylpyridine[3] were prepared analogously with the reported procedures. The synthetic procedure and characterization data of isotopically labeled 2-(Phenylethynyl)-1-([4-(4,4,5,5-tetramethyl-1,3,2-dioxaborolan-2-yl)phenyl]methyl-$d_2$)pyridin-1-ium-3,4,5,6-$d_4$-1-$^{15}$N bromide (**$^{15}$N-BBPEP**, which is the precursor of **$^{15}$N-BBSP**) were described in our previous paper.[4] Thin-layer chromatography (TLC) was performed on aluminum-backed TLC plates and compounds were visualized by UV light. Silica gel (230-400 mesh, 60 Å) was purchased from Sigma Aldrich and used for flash chromatography. $^{1}$H, $^{13}$C NMR spectra were recorded on Bruker Avance III HD spectrometers at 7.0 T. All coupling constants were measured in Hertz. All moisture sensitive reactions were carried out in oven-dried glassware using nitrogen industrial grade cylinders. High resolution mass spectra were recorded on a Thermo LTQ Orbitrap XL (ESI$^+$) or a P-SIMS-Gly of Bruker Daltonics Inc (EI$^+$).

### 3.2 Synthetic procedure (BBPEP and BBSP)

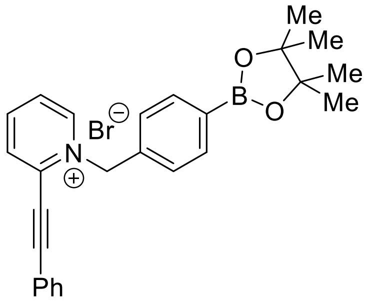

**2-(Phenylethynyl)-1-(4-(4,4,5,5-tetramethyl-1,3,2-dioxaborolan-2-yl)benzyl)pyridin-1-ium bromide ($^{14}$N-BBPEP)**

To a 15 mL Schlenk tube was added 2-(phenylethynyl)pyridine (170.0 mg, 0.95 mmol) and 2-(4-(bromomethyl)phenyl)-4,4,5,5-tetramethyl-1,3,2-dioxaborolane (282.1 mg, 0.95 mmol, 1.0 equiv) under a $N_2$ atmosphere. Then dry PhMe (4.0 mL) was added via canula. The mixture was heated at 90 ℃ for 16 h. At ambient temperature, cold $Et_2O$ (10.0 mL) was added and precipitate was formed. The precipitate was collected via filtration and it was washed with cold $Et_2O$ (2 × 1.0 mL) to afford the desired product (265.0 mg, 58%) as an off-white solid. **$^1$H NMR** (300 MHz, DMSO-*d*$_6$) $\delta$ = 9.28 (d, *J* = 6.2 Hz, 1H), 8.68 (dd, *J* = 7.9, 1.4 Hz, 1H), 8.43 (dd, *J* = 8.1, 1.5 Hz, 1H), 8.18 (ddd, *J* = 7.9, 6.1, 1.6 Hz, 1H), 7.78–7.48 (m, 6H), 7.42 (d, *J* = 7.7 Hz, 3H), 6.12 (s, 2H), 1.28 (s, 12H). **$^{13}$C NMR** (75 MHz, DMSO-*d*$_6$) $\delta$ = 147.51, 146.19, 137.44, 136.99, 135.42, 133.19, 132.88, 132.24, 129.57, 127.82, 119.27, 106.70, 84.34, 80.99, 62.96, 25.07. **HR-MS** (EI) *m/z* calcd for $C_{26}H_{27}BNO_2$ [M - Br]+ 396.2130, found 396.2133.

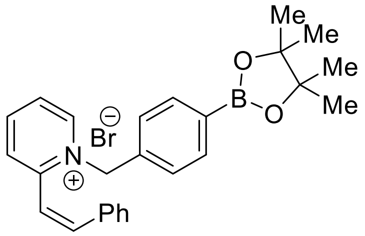


**(Z)-2-Styryl-1-(4-(4,4,5,5-tetramethyl-1,3,2-dioxaborolan-2-yl)benzyl)pyridin-1-ium bromide ($^{14}$N-BBSP)**

To a 15 mL Schlenk tube was added (Z)-2-styrylpyridine (100.0 mg, 0.55 mmol) and 2-(4-(bromomethyl)phenyl)-4,4,5,5-tetramethyl-1,3,2-dioxaborolane (163.6 mg, 0.55 mmol, 1.0 equiv) under a $N_2$ atmosphere. Then dry PhMe (3.0 mL) was added via canula. The mixture was heated at 90 ℃ for 16 h. At ambient temperature, cold $Et_2O$ (10.0 mL) was added and precipitate was formed. The precipitate was collected via filtration and it was washed with cold $Et_2O$ (2 × 1.0 mL) to afford the desired product (7.0 mg, 2.6%) as an off-white solid. **$^1$H NMR** (300 MHz, DMSO-*d*$_6$) $\delta$ = 9.18–9.15 (m, 1H), 8.64–8.59 (m, 2H), 8.07–8.02 (m, 1H), 7.89–7.84 (m, 1H), 7.77–7.59 (m, 5H), 7.51–7.46 (m, 3H), 7.32 (d, *J* = 7.6 Hz, 2H), 6.17 (s, 2H), 1.23 (s, 12H). **$^{13}$C NMR** (75 MHz, DMSO-*d*$_6$) $\delta$ = 152.66, 146.64, 145.73, 143.64, 137.80, 135.49, 135.10, 131.29, 129.54, 128.91, 127.26, 126.48, 126.29, 117.90, 84.28, 60.63, 25.07. **HR-MS** (EI) *m/z* calcd for $C_{26}H_{29}BNO_2$ $[M–Br]^+$ 398.2286, found 398.2286.

### 3.3 Reference

[1] N. Nishiwaki, S. Minakata, M. Komatsu, Y. Ohshiro, *Chem. Lett.* **1989**, *18*, 773-776.

[2] H. Park, J. Chen, I. E. Dimitrov, J. M. Park, Q. Wang, *ACS Sens.* **2022**, *7*, 2928-2933.

[3] Y. Chen, T. Jing, Z. Cai, Q. Bu, *Org. Biomol. Chem.* **2025**, *23*, 5307-5312.

[4] R. Mei, L. M. Fries, T. L. K. Hune, M. D. Santi, G. G. Rodriguez, S. Sternkopf, S. Glöggler, *Angew. Chem. Int. Ed.* **2024**, *63*, e202403144.

### 3.4 NMR and HR-MS Spectra

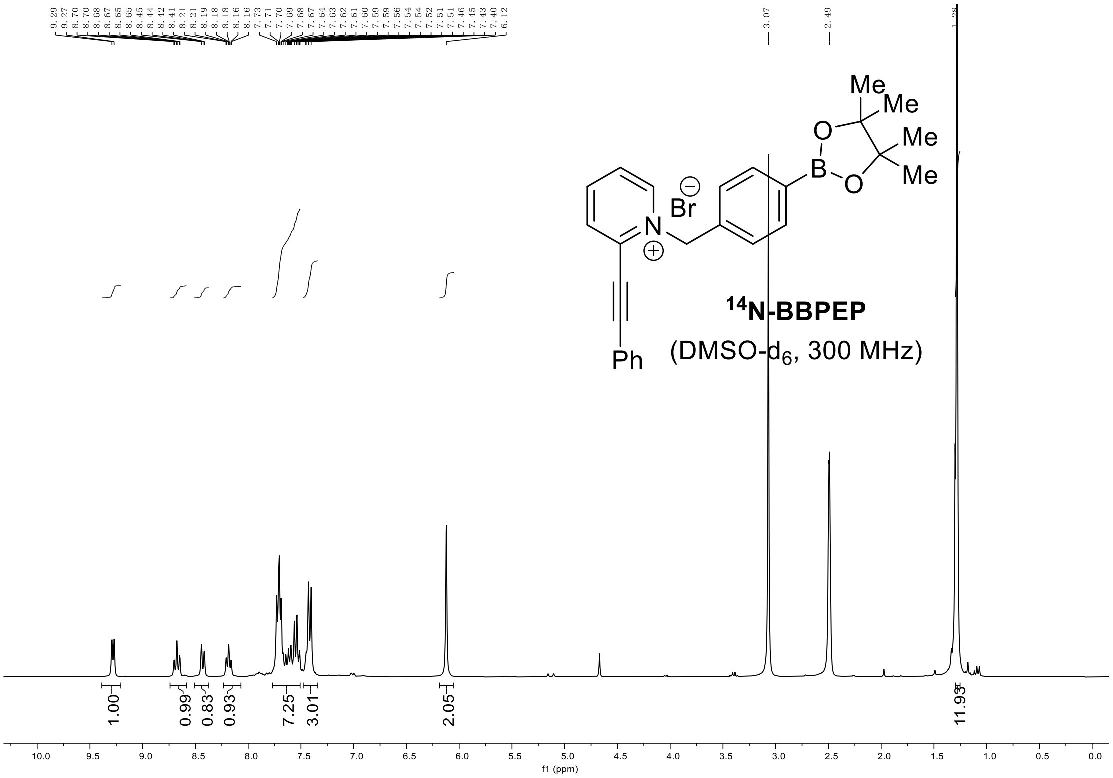


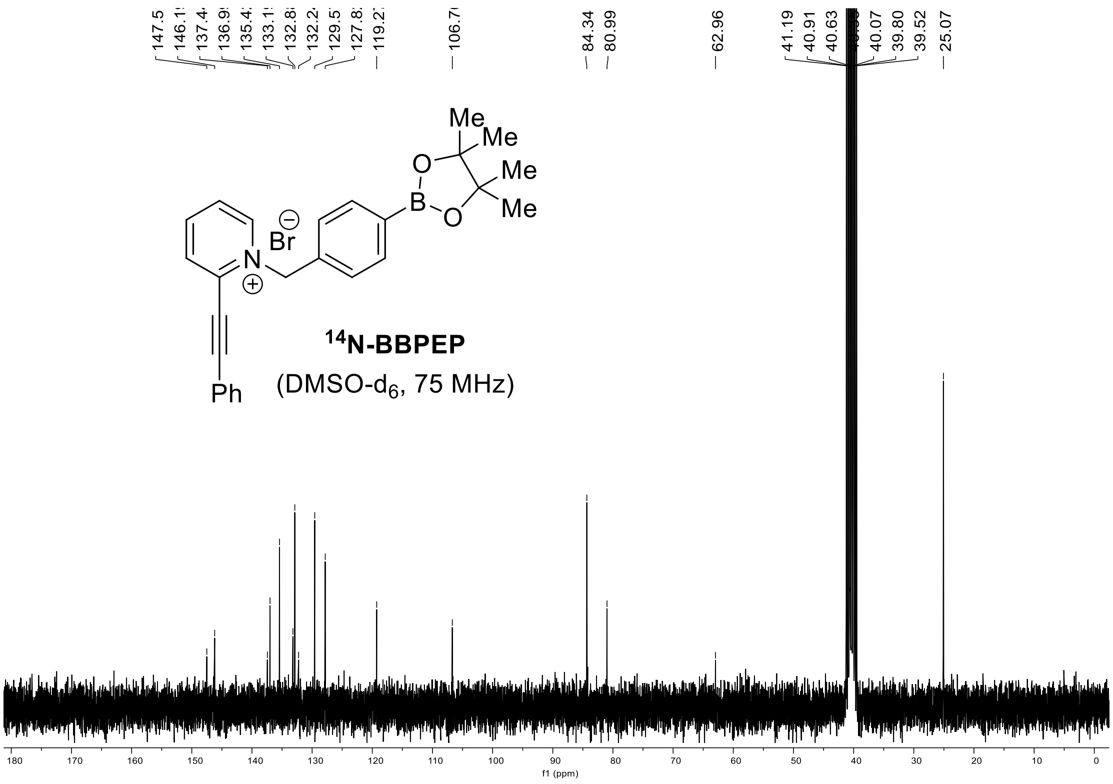

E:\2025\Oktober\161025\yli00205.raw 10/16/2025 7:00:10 AM

yli00205 #19-27 RT: 0.08-0.11 AV: 9 SB: 8 0.01-0.04 NL: 1.34E10
T: FTMS + p ESI Full ms [150.0000-2000.0000]

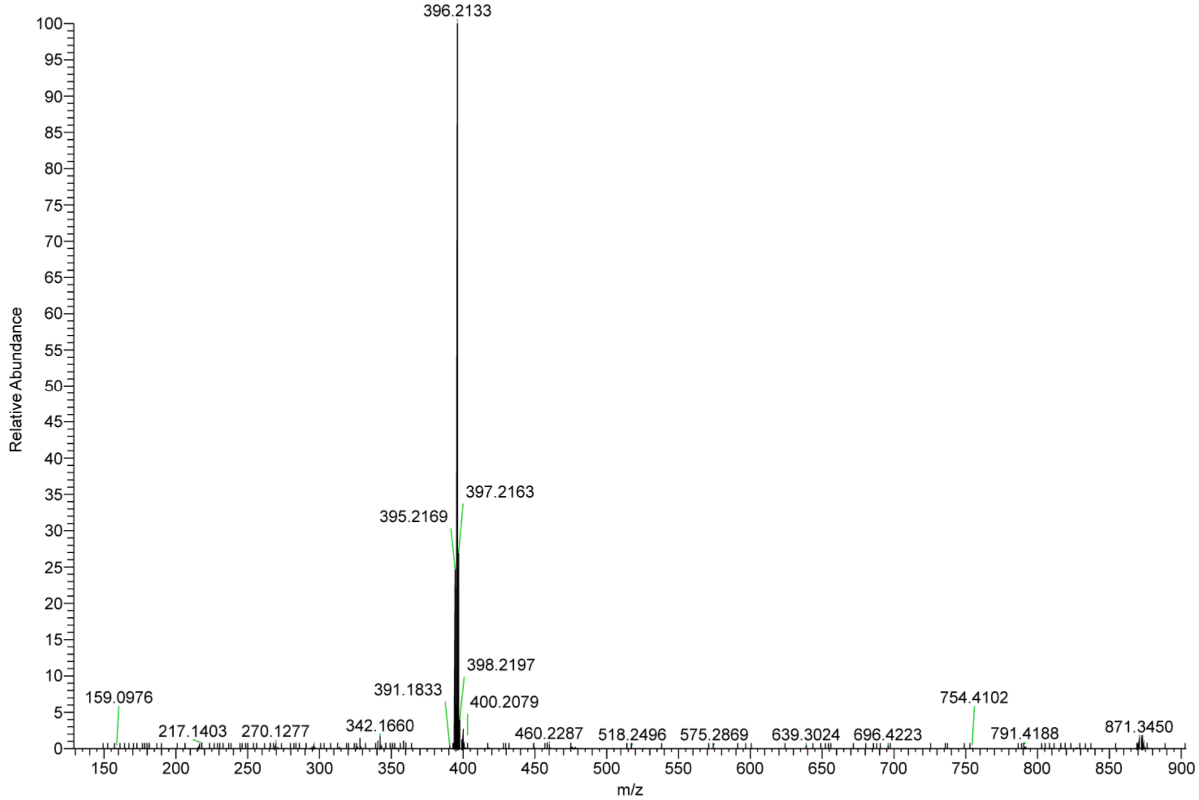


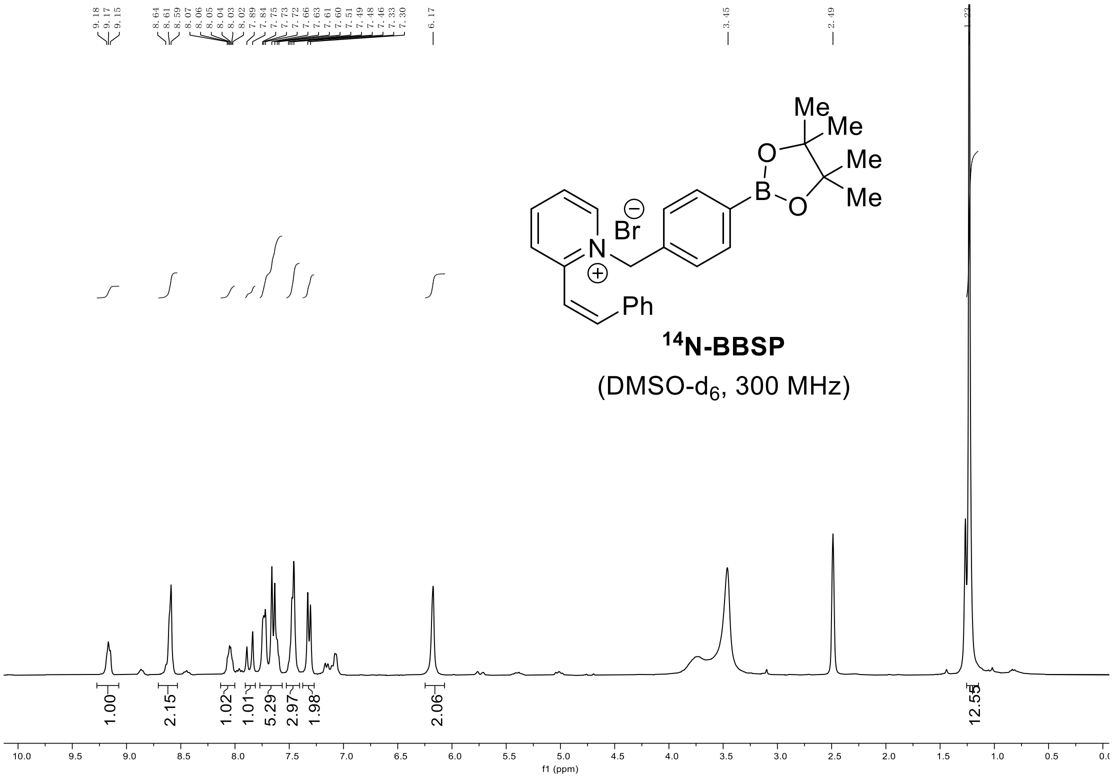

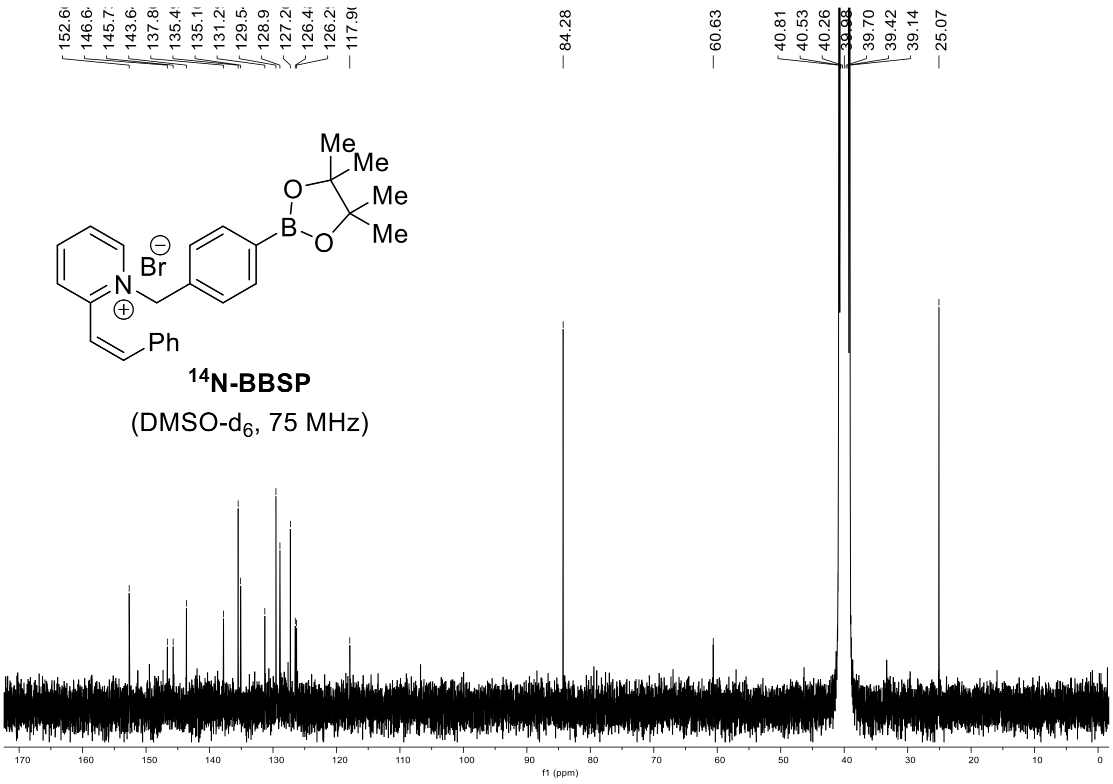

152.6
146.6
145.7
143.6
137.8
135.4
135.1
131.2
129.5
128.9
127.2
126.4
126.2
117.9
84.28
60.63
40.81
40.53
40.26
39.98
39.70
39.42
39.14
25.07
Me
Me
Me
Me
O
B
O
Br
N
Ph
14N-BBSP
(DMSO-d6, 75 MHz)
170 160 150 140 130 120 110 100 90 80 70 60 50 40 30 20 10 0
f1 (ppm)

E:\2025\Oktober\161025\yli00204.raw 10/16/2025 6:55:32 AM

yli00204 #19-25 RT: 0.08-0.11 AV: 7 SB: 8 0.01-0.04 NL: 3.45E9
T: FTMS + p ESI Full ms [150.0000-2000.0000]

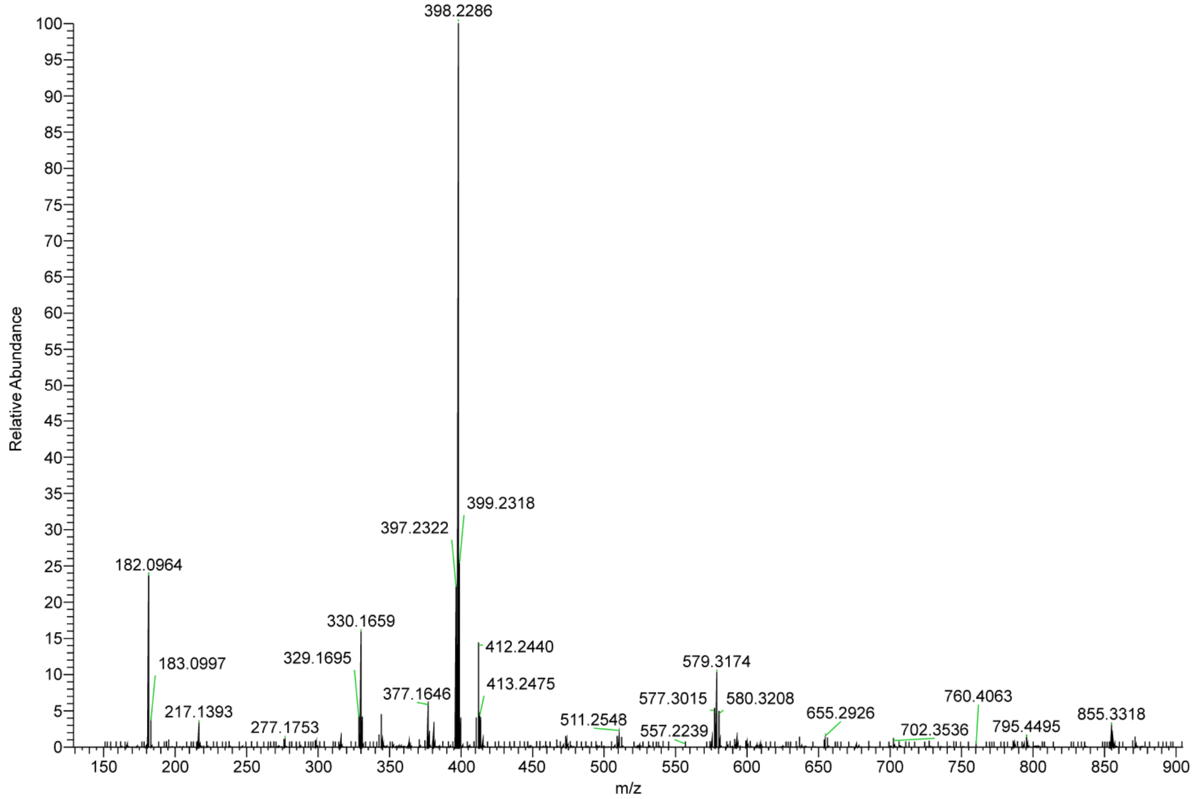

Supplement: Supplementary file 1 — Supporting Information [file CHEM-31-e03018-s001.pdf]
